# Viscosity Flow Curves of Agar and the *Bounded Ripening Growth* Model of the Gelation Onset

**DOI:** 10.3390/molecules29061293

**Published:** 2024-03-14

**Authors:** Vincenzo Villani

**Affiliations:** Dipartimento di Scienze, Università della Basilicata, 85100 Potenza, Italy; vincenzo.villani@unibas.it

**Keywords:** gelation, gelation onset, hydrogels, Weber number, logistic equation, agar solution, Ostwald ripening, rotating liquid drops

## Abstract

The gelation kinetics of agar aqueous solutions were studied by means of the viscosity flow curves using a coaxial Couette cylinder viscometer. The viscosity curves show an unusual sigmoidal trend or an exponential decay to a viscous steady state. An original theory of gelation kinetics was developed considering the coarsening of increasingly larger and more stable clusters due to Ostwald ripening and the breakup of clusters that were too large due to the instability of rotating large particles induced by the shear rate. The developed *Bounded Ripening Growth* model takes into account the trend of the viscosity curves by means of an autocatalytic process with negative feedback on aggregation according to the logistic kinetic equation, in which the constants $k_{1}(\gamma )$ and $k_{-}(\nu )$ are governed by the surface tension and shear rate, respectively. A dimensionless equation based on the difference between the Weber number and the ratio of the inverse kinetic constant to forward constant, accounts for the behavior of the dispersed phase in equilibrium conditions or far from the hydrostatic equilibrium.

## 1. Introduction

The gelation (or *sol-gel* transition) of polymer solutions is a hot topic of scientific and technological interest [1]. In particular, the preparation of hydrogels is important in the food, cosmetic, pharmaceutical, medical and bioplastics industries; furthermore, it is fundamental in the fabrication of 3D scaffolds for tissue engineering [2,3,4,5]. From the rheological point of view there are many open problems, such as the optimization of the formulation, the control of the aggregation kinetics and the crosslinking of the polymer material [6].

The onset of gelation arises from the formation of a transient fluctuating network of particles interacting reversibly through short-range attractive interactions lightly exceeding the thermal energy *kT*, as in the Baxter sticky sphere model [7]. The average cluster size diverges strongly with time near gelation and, in an experimental system, the position of the gel point is associated with the inflection point on the viscosity flow curve or a substantial increase in the high-frequency storage modulus [8].

In this paper, the gelation onset of agar polymer solutions was studied by determining the viscosity curves as a function of time, η=η(t;ν,c,T), using a Couette coaxial cylinder rotational viscometer. The shear rate ν, concentration c, temperature T and rheological history during experiments were varied. The viscometry method has been widely used in the determination of the gelation time from the inflection point on the viscosity flow curve [9].

In general, the coarsening kinetics of dispersed clusters is given by the Ostwald ripening [10] according to the Voorhees equation [11]:

$$4\pi R^{2}\frac{dR}{dt}=\mu \cdot J$$

$$J=-D\oint_{S}\nabla c\cdot dS$$

The equation represents the flow of matter across the surface cluster of radius R according to the first Fick equation, where μ is the molar volume of the aggregate and J is the diffusion flux integral. The equation is valid for both growing or dissolving particles, giving rise to an exponential growth of the aggregate phase at the expense of the smaller particles:

$$R^{3}=R_{0}^{3}\cdot \exp(k\cdot t)$$

The concentration B of the dispersed particles is given by the Kelvin equation [12], where $B_{\infty }$ is the equilibrium concentration of the gel state and γ is the surface tension:

$$B(R)=B_{\infty }\cdot \exp(\frac{2\gamma \cdot \mu }{kT\cdot R})$$

The evolution of the particle size distribution function f(t,R) is given by the partial differential equation of Alexandrov and Alexandrova [13], where D is the diffusion coefficient of particles:

$$\frac{\partial f(R,t)}{\partial t}+\frac{\partial }{\partial R}(\frac{dR}{dt}\cdot f)=\frac{\partial }{\partial R}(D\cdot f)$$

These theories take into account the asymptotic state of gelation process when larger clusters growth at the expense of smaller ones in an unbounded way.

In this work, an original coarsening model, which we call the *Bounded Ripening Growth* (BRG) of the aggregation of elementary particles at the onset of gelation has been developed, taking into account the surface tension of the dispersed particles (Laplace’s equation), the instability of rotating liquid drops (Brown’s equation) and the diffusive processes (Fick’s first law) in which the larger and more stable particles grow at the expense of the smaller and more unstable particles, which dissolve in the elementary particles and diffuse below the action of the concentration gradient (Ostwald ripening). The dispersion viscosity is a linear function of the volume fraction of the dispersed phase according to the Einstein equation. 

A logistic kinetics equation is obtained as a function of the concentration of the dispersed phase, in which the forward constant is controlled by the surface tension and the inverse constant by the applied shear rate.

## 2. Material and Methods Section

The aqueous solutions of agar (food additive E406) at 1 and 1.5% by weight are prepared at a temperature of 80 °C. Furthermore, the blend agar 1%–hyaluronic acid 0.5% (aqueous solution of sodium hyaluronate 1%) was considered.

The cooling of the samples to the target values of 60, 50, 45 and 40 °C was accomplished by coupling the Couette cell of the viscosimeter (ViscoQC Anton Paar, Anton Paar, Graz, Austria) to a heated and refrigerated circulating bath (Haake DC30, Thermo Haake, Karlsruhe, Germany). Viscosity flow curves of 1 h were collected with a time step of 30 s in the viscosity range of 100 mPa s. The rotational frequency of 60 or 30 rpm (revolutions per minute) was applied.

## 3. Experimental Section

**Figure 1:**
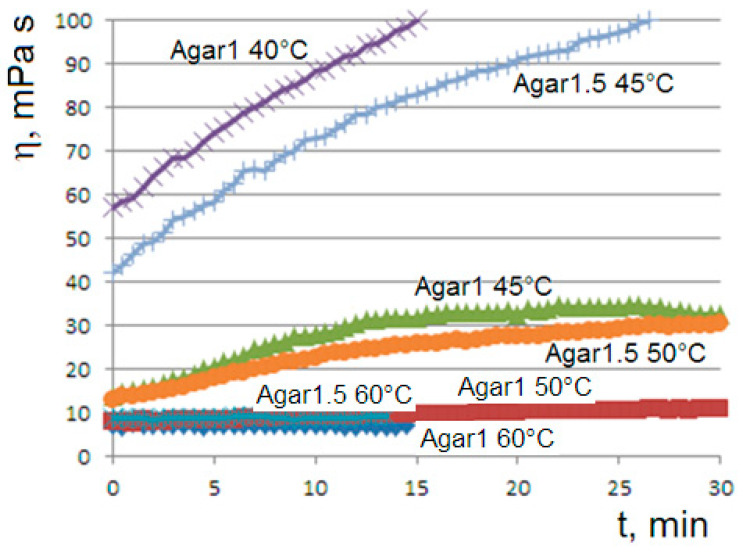
Viscosity flow curves at 60, 50, 45 and 40 °C for the 1% agar solution (*Agar*1 in labels) and at 60, 50 and 45 °C for the 1.5% agar (*Agar*1.5 in labels). The applied rotational speed is 60 rpm.

When the aqueous solutions of agar at 1% or 1.5% are cooled to values of 45 or 50 °C, we observe the onset of the gelation transition, with a higher temperature for the more concentrated solution. At the gelling point, the solution becomes heterogeneous with the formation of dispersed aggregates, which gradually become larger until the formation of the gel phase, corresponding to a connected agarose chains network. The gelation kinetics are monitored via the viscosity flow curves η=η(t). At the onset temperatures (45 °C for agar at 1% and 50 °C for agar at 1.5%), the curves show the appearance of an unusual sigmoidal trend towards a viscous steady state (Figure 1).

**Figure 2:**
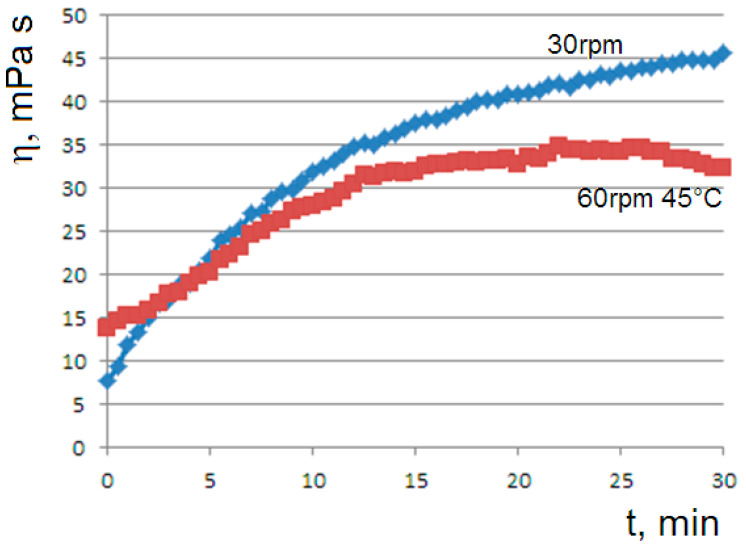
Viscosity flow curves at 45 °C for the 1% agar solution at rotational speeds of 60 (blue line) or 30 rpm (red).

The dependence of the gelation kinetics on the shear rate was highlighted by the means of the viscosity curves at different rotational speeds; by increasing the shear rate, the achievement of the steady state in the gelation process is favored (Figure 2).

**Figure 3:**
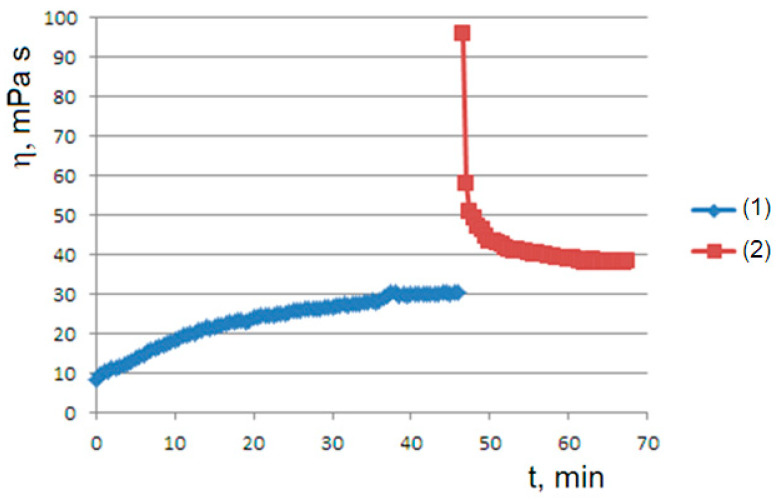
The rheological history experiment of flow–pause–flow: the initial viscosity curve (1) is followed by a 60 min pause and then by a final viscosity curve (2). An agar solution at 1% and a rotational speed of 60 rpm are used.

The role played by the shear rate is demonstrated by the viscosity graph obtained after a pause in flow for a sufficiently long time (45 min): the flow–pause–flow experiment. In this first rheological history experiment, when the test resumes after the pause, the viscosity starts from high values due to the formation of the gel state. However, the viscosity rapidly decays, due to the applied shear rate, towards the viscous steady state observed in the initial curve (Figure 3). This behavior is explained later by the proposed model.

**Figure 4:**
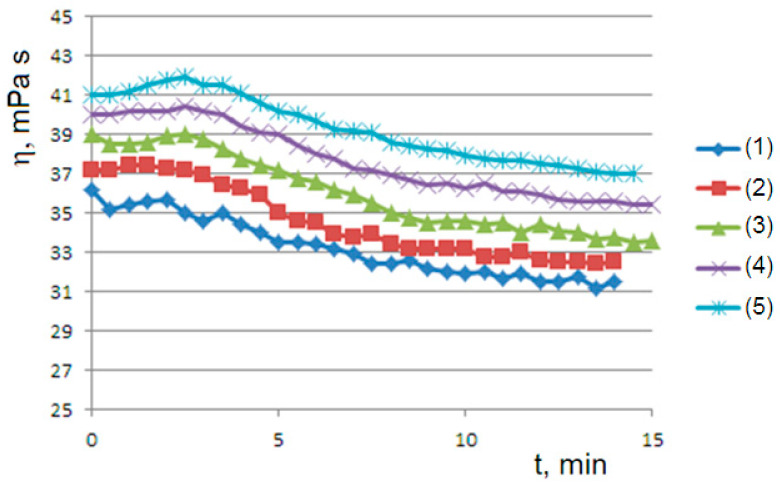
The rheological history experiment of gelling–mixing–flow: a succession of viscosity curves (from (1) to (5)) from the bottom to top at 45 °C with homogenization of the sample at the end of each test. An agar solution at 1% and rotational speed of 60 rpm are used.

**Figure 5:**
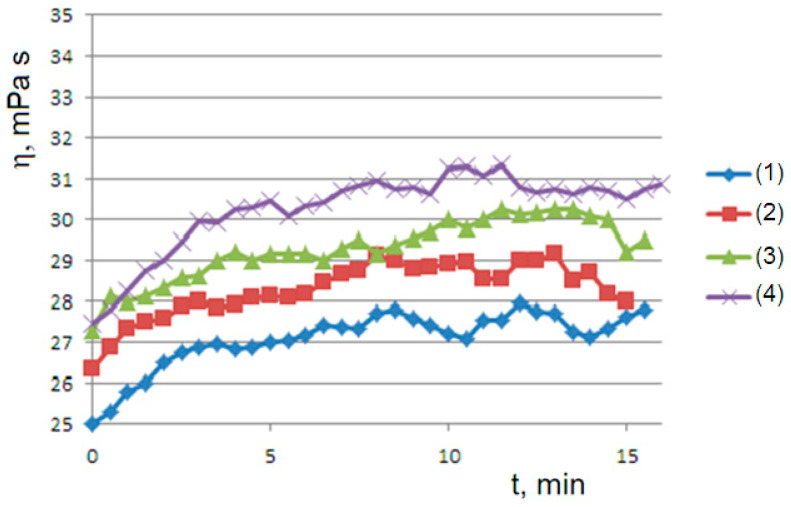
The rheological history experiment of gelling–mixing–flow: a succession of viscosity curves (from (1) to (4)) from the bottom to top at 50 °C with homogenization of the sample at the end of each test. An agar solution at 1% and rotational speed of 60 rpm are used.

A second rheological history experiment of gelling–mixing–flow was carried out using a succession of viscometric curves interspersed with the mechanical homogenization of the sample. At 45 °C for the 1% sample (Figure 4), we obtain a series of curves that relax from top to bottom to steady states, highlighting that mechanical stirring has produced an initial concentration of dispersed phase greater than the final concentration $B_{0}>B_{\infty }$ (supersaturated state). On the contrary, at 50 °C for the 1.5% sample (Figure 5), we obtain a series of curves that converge from bottom to top to steady states; this is consistent with an initial concentration of the dispersed phase that is lower than the final concentration $B_{0}<B_{\infty }$ (undersaturated state), which occurs at a higher temperature. Furthermore, in both cases, we observe convergence to progressively higher values of equilibrium concentration $B_{\infty }$ by iterating the stirring–gelling cycle. In all cases, as we will see, the observed curves are consistent with the *Bounded Ripening Growth* model proposed in this work.

**Figure 6:**
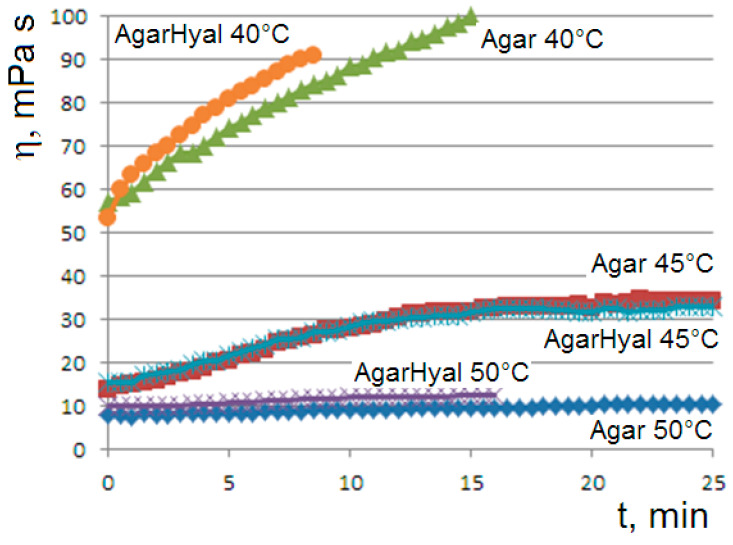
Viscosity curves at 50, 45 and 40 °C (decreasing from the bottom to top) for the blend (agar 1%–hyaluronic acid 0.5% aqueous solution) (*AgarHyal* in the label) *vs* the agar 1% reference solution (*Agar* in the label). The applied rotational speed is 60 rpm.

The trend of the viscosity curves is confirmed in two-component hydrogels. Figure 6 shows the curves of the blend agar 1%–hyaluronic acid 0.5% *versus* the agar 1% reference solution at temperatures from 60 to 40 °C. At 45 °C, the sigmoidal curves of the two systems are perfectly overlapped, indicating that the long hyaluronic chains adsorbed on the agarose network do not interfere in the clusters coarsening.

## 4. Theoretical Section

### 4.1. Bounded Ripening Growth Model

The *Bounded Ripening Growth* model of gelation is developed, taking into account the growth of aggregates due to the driving force of surface tension according to Ostwald ripening and the breakup of aggregates via negative shear rate feedback, due to the instability of clusters that are too large. Finally, the viscous steady state given by the formation–destruction equilibrium of the aggregates is reached [14,15,16].

The agarose macromolecules at a temperature of about 50 °C (depending on the concentration of the aqueous solution) couple (coil-to-helix transition), giving rise to the double helix elementary sol particles A [17]. The double helices have pending chains that allow the formation of increasingly large clusters B, according to exponential coarsening.

Let us consider the proposed *Bounded Ripening Growth* model. In the first step of gelation, the minimization of the surface free energy of the system prevails, which favors the growth of the clusters. For simplicity, we assume Laplace’s law for spherical particles dispersed in the continuous medium (1), where Δp is the Laplace pressure across the particle surface:

$$\Delta p=\frac{2\cdot \gamma }{R}$$

**Figure 7:**
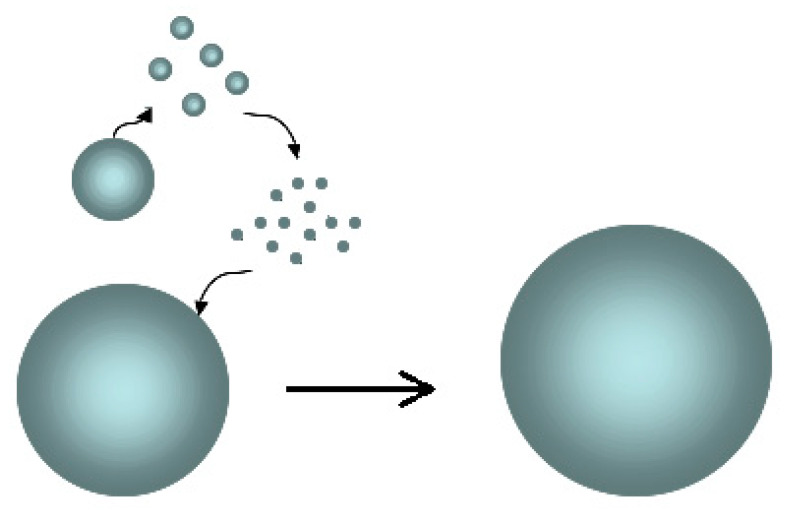
Picture of the Ostwald ripening mechanism: the small particles dissolve in the constituent elements which re-aggregate on the larger particles, determining their unbounded growth.

The *Ostwald ripening* mechanism is the basis of the increase in the size of the dispersed particles (2) (Figure 7).

The smaller *sol* aggregates tend to spontaneously dissolve (the feedforward effect) due to the high Laplace pressure and low surface tension, freeing the elementary particles; therefore, the free particles spread in the aqueous medium towards the larger clusters and down the concentration gradient in accordance with Fick’s first Law; finally, their adsorption reduces the interfacial energy of the system.

In general, the viscosity of dispersions is governed by the Einstein equation

$$\eta (\phi )=\eta_{0}(1+\frac{5}{2}\cdot \phi )$$

where $\eta_{0}$ is the viscosity of the continuous phase, in which the aggregates are dispersed, and ϕ is the volume fraction of the dispersed phase.

The volume fraction of the aggregates is given by

$$\phi =\frac{V_{B}}{V}$$

where $V_{B}$ is the volume of the dispersed phase and V is the total volume of the dispersion. Therefore, the *volume fraction* is proportional to the *concentration* of the dispersed phase and, assuming the unit density, coincide:

$$\phi =\frac{V_{B}}{V}=\frac{\rho M_{B}}{V}=\rho \cdot B=B$$

The BRG model consists of three steps depending on the degree of the coarsening progress. For still-small aggregates, the unbounded growth aggregation mechanism (Ostwald’s ripening) prevails (1); when the aggregates have become sufficiently large, the negative feedback is triggered the breakup of the clusters due to applied shear rate (2), and the elementary particles adsorbed by the growing clusters are released. Then, the loose particles aggregate again, and so on. Finally, a steady state is reached in which the processes of formation (the feedforward of the surface tension) and destruction (the negative feedback of the shear rate) reach equilibrium (3). The result, as we will see, is an asymptotic plateau trend of the cluster concentration in agreement with the observed sigmoidal viscosimetric curves.

In the initial ripening step, we represent the gelation process by an autocatalytic process, in which the elementary particle A “reacts” with the cluster $B_{n}$ to give the cluster larger by one unit $B_{n+1}$ in the coarsening process:

$$A+B_{n}\to B_{n+1}$$

**Figure 8:**
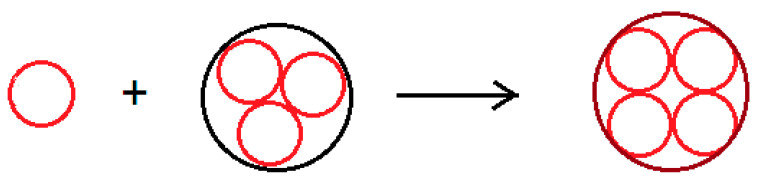
Picture of the autocatalytic process $A+B_{n}\to B_{n+1}$.

Figure 8 schematizes the autocatalytic process in which we assume the concentration of the species A is constant.

This results in the first order kinetic equation are written as

$$\frac{dB}{dt}=k_{1}\cdot B$$

The solution of which is the exponential growth of the concentration of the species B over time:

$$B(t)=B_{0}\cdot \exp(k_{1}\cdot t)$$

According to Einstein’s equation, the viscosity of the dispersion also varies exponentially:

$$\eta (t)=\eta_{0}(1+k_{E}\cdot B(t))$$

### 4.2. Instability of the Clusters

During the viscometric experiment, the shear stress τ on the fluid is proportional to the shear rate ν of the fluid according to Newton’s law:

$$\tau =\eta \cdot \frac{du}{dt}=\eta \cdot \nu$$

where u is the linear flow rate. 

Due to the shear rate, the dispersed aggregates experience a mechanical moment that keeps the particles rotating around the axis, passing through the center of gravity and perpendicular to the flow direction (vorticity axis); the instability of liquid rotating particles is the basis of the breakup mechanism of dispersed clusters that are too large.

The dynamics of an isolated rotating particle in the flow field was first addressed by Einstein [18] for the case of a newtonian medium. Under steady low shear, the sphere translates in the flow direction, while rotating around the vorticity axis. Thus, in a frame translating with the sphere center, the sphere just rotates in time with a constant angular velocity ω [19]. 

Einstein, and subsequently Jeffery [20], demonstrated that under no-slip boundary conditions at the particle surface, the angular velocity is independent of particle size $\omega =\frac{\nu }{2}$. Furthermore, the rotating spherical particle of radius R experiences a centrifugal force (gyrostatic pressure) with a magnitude at the surface dependent on the size and angular velocity $F_{c}=m\omega^{2}R$.

Plateau [21] found that as the angular velocity increased, a liquid sphere suspended in a liquid medium progressed through a sequence of shapes which evolve to ellipsoidal and lobed shapes and, finally, breakup. These experimental works have been repeated with basically the same outcome [22,23].

The equilibrium shapes of a rotating drop held together by surface tension are governed by the Brown and Scriven nonlinear differential equation [24]:

$$2\gamma \cdot \nabla^{2}R(r,\vartheta ,\phi )=\Delta p+\frac{1}{2}\omega^{2}r^{2}\cos^{2}\vartheta \cdot \Delta \rho$$

R(r,ϑ,ϕ) is the surface of the particle represented by the surface vector $\vec{R}$ in spherical coordinates; γ is the surface tension; Δρ is the density difference between the liquid of the drop and the surrounding fluid; ω is angular velocity of the drop; rcos(ϑ) is the perpendicular distance from a point on its surface to the axis of rotation; and Δp is the Laplace pressure across the drop surface. 

As the angular velocity increases, the particle surface vector R(r,ϑ,ϕ) progresses through a sequence of shapes, ellipsoidal axisymmetric at first, then no-axisymmetric and lobed shape. The no-axisymmetric shapes are stable at low rotational rates but lose stability at the bifurcation to lobed drops. Then, we have two lobed shapes: the symmetrical Darwin’s dumbbell shape [25] or the asimmetrical Poincarè pear shape [26]. In any case, they undergo fission into two ovoid particles of the same or different size [27].

**Figure 9:**
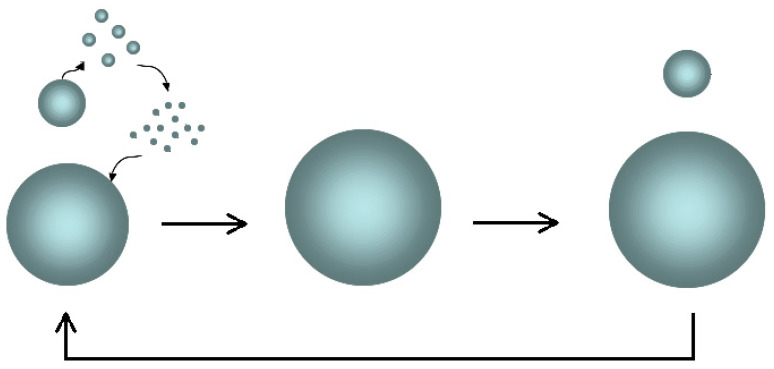
Picture of the autocatalytic mechanism of *Bounded Ripening Growth* in which the unbounded growth of Ostwald ripening is bounded by the fission of larger aggregates.

What happens as the size of the rotating particles increases while keeping the angular velocity constant? This is what concerns the ultimate fate of the particles in our dispersion. The aggregates all rotate with the same angular velocity; however, the angular momentum is greater for larger particles. This means that the instability of the larger aggregates is favored for the same angular rotation. By means of the rotation of the dispersed aggregates and by the low surface tension of the aggregates, they undergo beakup at a critical dimension, thus, establishing a negative feedbeak loop to the formation of larger aggregates. On the other hand, the particles that are too small are unstable at Laplace pressure and dissolve into the constituent particles. The free elementary particles reaggregate according to the Ostwald mechanism and fuel the equilibrium of the *Bounded Ripening Growth* (Figure 9).

### 4.3. Kinetic Equation of the Bounded Ripening Growth 

**Figure 10:**
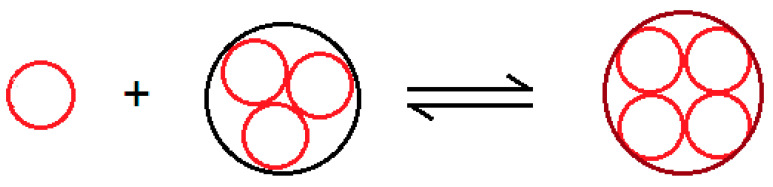
Picture of the bounded autocatalytic process $A+B_{n}↔B_{n+1}$.

When the aggregates break down, a reverse process with negative feedback overlaps with the direct formation process with the feedforward effect. In this way, the exponential growth is replaced by bounded growth kinetics of the logistic type, according to the equilibrium equation (Figure 10)

$$A+B_{n}↔B_{n+1}$$

The kinetic constant becomes dependent on the concentration of the clusters B, on the forward $k_{1}$ and reverse $k_{-}$ kinetic constants:

$$k(t)=k_{1}-k_{-}\cdot B(t)$$

This gives rise to the logistic kinetic equation with the constants controlled by surface tension $k_{1}(\gamma )$ and shear rate $k_{-}(\nu )$:

$$\frac{dB}{dt}=(k_{1}-k_{-}\cdot B)\cdot B$$

$$\frac{dB}{dt}=k_{1}(\gamma )\cdot B-k_{-}(\nu )\cdot B^{2}$$

The logistic non-linear differential equation admits the sigmoid function (or sigmoidal curve) B(t) as the analytical solution:

$$B(t)=\frac{B_{\infty }}{1+\frac{B_{\infty }-B_{0}}{B_{0}}\cdot \exp(-k_{1}\cdot t)}$$

where $k_{1}$ incorporates the concentration of the species A, considered constant, and $B_{\infty }$ is the concentration at equilibrium:

$$B_{\infty }=\frac{k_{1}}{k_{-}}=\frac{k_{+}\cdot A_{0}}{k_{-}}$$

**Figure 11:**
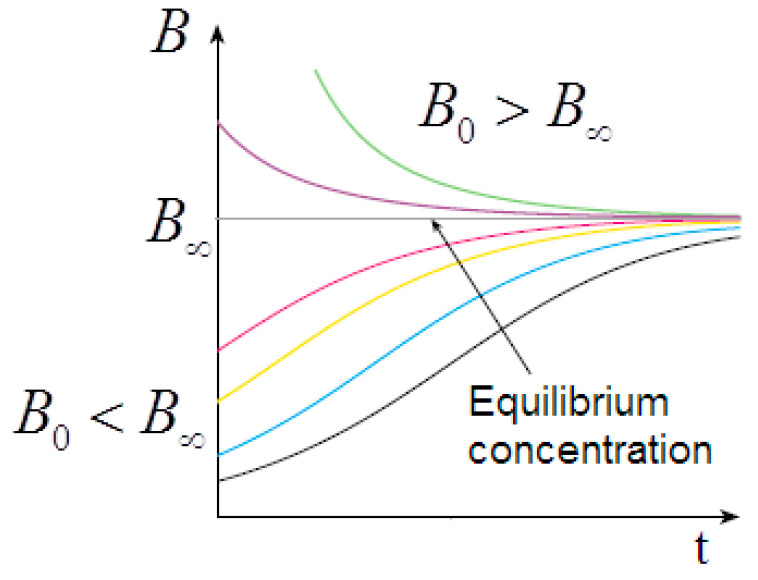
Solutions of the logistic equation: sigmoidal trend for $B_{0}<B_{\infty }$ or exponential decay for $B_{0}>B_{\infty }$.

The solution depends on the initial conditions. For $B_{0}<B_{\infty }$, we have a sigmoidal trend of B(t) and, consequently, of η(t); however, for $B_{0}>B_{\infty }$ we have an exponential decay to the equilibrium value in excellent agreement with the observed viscosity curves (Figure 11).

The viscosity curve of the sample homogenized at 45 °C presents the typical relaxation of a supersaturated solution in agreement with the proposed logistic model. However, the kinetics converge to increasingly higher equilibrium concentrations by iterating the homogenization treatment.

Homogenizing the material at 50 °C, we observe the characteristic viscosity curves of an undersaturated solution, with a progressive increase in the equilibrium concentration by iterating the treatment.

Therefore, the cluster disaggregation modifies the final equilibrium $B_{\infty }$ by varying the initial concentration $A_{0}$ of the free species by increasing its concentration at 45 °C or decreasing it at 50 °C. 

### 4.4. Dimensionless Sol-Gel Equilibrium Equation

At hydrostatic equilibrium, the Laplace pressure and the shear stress in the Newtonian flow give the constitutive equation:

$$\eta \nu -\frac{2\gamma }{R}=0$$

Then,

$$We=\frac{R\cdot \eta \nu }{2\gamma }=1$$

where the *Weber number* We is defined by the ratio of shear stress to surface tension [28]. At steady state, the creation–destruction processes balance and the Weber number is unity:

We−1=0

On the contrary, far from hydrostatic equilibrium, in conditions in which surface tension and, therefore, the creation process prevails (at low shear rates or for small particles), we have

We<0

At enough high shear rates or particle sizes, the destruction process prevails, and we have

We>0

If J is the flow of particles then $J_{+}$ and $J_{-}$ are the transport of matter into the cluster or out of the cluster, respectively, where the positive flow is representative of aggregation; negative flow is the disaggregation of the clusters; and zero is the steady state. For We>1, dispersed particles undergo breakup with the release of small particles.

The set of constitutive equations and inequations of the Weber number

We−1=0        We−1<0        We−1>0

are reduced to a single dimensionless equation based on the difference between the Weber number and the number Wi:

We−Wi=0

where Wi is given by

$$Wi=\frac{J_{-}}{J_{+}}=\frac{k_{-}}{k_{+}}=\frac{1}{K}=\frac{A_{0}}{B_{\infty }}$$

where K is the equilibrium constant of reaction.

## 5. Discussion Section: Experiments vs. Theory

Consistent with the viscosity curve of Figure 1, the *Bounded Ripening Growth* predicts a sigmoidal trend in the concentration of the dispersed phase during the gelation. Consistent with the solutions of the logistic equation of Figure 11, the sigmoidal trend depends significantly on the initial concentration of the dispersed particles. 

The negative feedback due to the shear rate as predicted by the model is consistent with the viscosity curves of Figure 2; as the shear rate increases, the viscosity is reduced and the final steady state is favored.

The solutions of the logistic equation account for the observed rheological histories. They depend on the initial conditions for $B_{0}<B_{\infty }$ (undersaturated dispersion), where we have a sigmoidal trend of predicted B(t) and observed η(t); for $B_{0}>B_{\infty }$ (supersaturated dispersion), we have an exponential decay to the equilibrium value. A supersaturated state occurs in the flow–pause–flow experiment of Figure 3; during the pause, the concentration of the dispersed phase increases in an unbounded way, and the expected exponential decay is observed in the final flow curve. Furthermore, the expected trends are observed in the flow curves of Figure 4 and Figure 5, which run from the undersaturated state at 45 °C or from the supersaturated state at 50 °C after homogenization of the clusters. Furthermore, the kinetics converge to increasingly higher equilibrium concentrations in both cases in agreement with the proposed model: the disaggregation of the hydrogel modifies the final equilibrium $B_{\infty }$ values depending on the concentration of the free species $A_{0}$, which is increased in both cases due to the homogenization process.

The gelation of the two-component agarose-hyaluronic chains of Figure 6 is consistent with the gelation logistic kinetics. The deep compatibility of these systems of increasing complexity is of technological interest.

The microscopic mechanism of *Bounded Ripening Growth* is the basis of macroscopic logistic kinetics, and the low surface tension of the particles of growing hydrogels accounts for the instability of the large particles at the applied shear rates.

The determination of the kinetic constants of the logistic equation that governs the gelation kinetics and the extension of the theory to further and more complex experimental cases are the subject of ongoing work.

The study of viscosity flow curves allows determining the onset of the sol-gel transition in a simple way through the appearance of the characteristic viscous steady state: this may have useful real-life applications. The behavior is viscous, as it is related to the formation of a fluid dispersion in which the particles are isolated and do not form a connected network. At temperatures lower than the onset value, the formation of an extended network finally prevails and the transition from the viscous dispersion to the viscoelastic gel state occurs, whose behavior is being studied using viscoelastic characterization according to loss and storage moduli.

## 6. Conclusions

The complexity of the gelation kinetics of aqueous agarose-based solutions was addressed from a viscometric and theoretical point of view. The sigmoidal trend of the viscosity curves as a function of time and temperature, the dependence of the trend of the curves on the applied shear rate and rheological histories were highlighted. The kinetics of the transition were described using an autocatalytic process with negative feedback. A logistic equation was obtained as a function of the concentration of the dispersed phase, in which the forward constant is governed by the surface tension of the dispersed particles, and the inverse constant is governed by the applied shear rate:

$$\frac{\partial B}{\partial t}=k_{1}(\gamma )\cdot B-k_{-}(\nu )\cdot B^{2}$$

The solutions of the logistic equation account for the trend in the sigmoidal or exponential decay viscosity curves.

The gelation process, at equilibrium and far from equilibrium, is controlled by the dimensionless equation

We−Wi=0

in which the hydrostatic balance is given by the difference between the Weber number We and the dimensionless ratio of the inverse kinetic constant Wi.

The driving force of the aggregation process is due to the minimization of the surface tension of the dispersed phase according to the Ostwald ripening. However, an antagonistic factor is due to the instability of enough large aggregates that deform and eventually breakup, releasing small aggregates that are unstable from the point of view of the surface tension. Thus, unstable large aggregates to the shear rate represent the bounding growth factor, and unstable small aggregates to the surface tension is the aggregate growth factor. In this way, a viscous steady state is due to small particle ripening and disaggregation due to the size instability of the clusters that are too large.

In general, Ostwald ripening is the microscopic process underlying the autocatalytic process with indefinitely growing matter transport, described by a first-order kinetic equation. However, as in our case, negative feedback is present, a bounded autocatalytic process results, and given by the microscopic process of bounded ripening growth, the observed logistic kinetics corresponds to this microscopic process. 

The complete description of the *Bounded Ripening Growth* at the microscopic scale requires a system of differential equations of Ostwald ripening and the Laplace equation of rotating particles. To take into account the deformation of the spherical particles and the diffusion-controlled growth process, it is necessary to consider equations in which the particle surface vector is time dependent R(r,ϑ,ϕ,t). Therefore, we must write

$$2\gamma \cdot \nabla^{2}R(r,\vartheta ,\phi ,t)=\Delta p+\frac{1}{2}\omega^{2}r^{2}\cos^{2}\vartheta \cdot \Delta \rho$$

$$\frac{\partial R(r,\vartheta ,\phi ,t)}{\partial t}=\frac{\nu }{4\pi R^{2}}D\oint_{S}\nabla c\cdot dS$$

We have a complex system of partial differential equations whose analytical and numerical solutions are the subject of a work in progress.

## Data Availability

Data are contained within the article.
